# Assessment of the Value of Routine Blood Cultures in the Evaluation and Treatment of Patients With Chorioamnionitis

**DOI:** 10.1155/S1064744994000487

**Published:** 1994

**Authors:** Gregory J. Locksmith, Patrick Duff

**Affiliations:** Division of Maternal-Fetal Medicine Department of Obstetrics and Gynecology University of Florida College of Medicine P.O. Box 100294 Gainesville FL 32610-0294 USA

## Abstract

*Objective:* The objective of this investigation was to determine the usefulness of blood cultures in evaluating patients with chorioamnionitis who were treated in accordance with a specific antibiotic protocol.

*Methods:* We reviewed the records of 539 patients with chorioamnionitis who delivered at our facility over a 3 year period (July 1, 1989–June 30, 1992). Patients had one set of aerobic and anaerobic blood cultures at the time of their initial assessment. They were treated initially with ampicillin or vancomycin plus gentamicin. Those who required cesarean delivery also received clindamycin postoperatively. Patients who had a poor initial response to therapy were treated empirically with selected antibiotics targeted against likely resistant organisms until the results of bacteriologic cultures were available. Bacteremic patients had repeat blood cultures while on therapy. We analyzed the medical records to determine the frequency with which blood culture results led to meaningful changes in patient management. We also compared the duration of febrile morbidity in bacteremic vs. nonbacteremic patients.

*Results:* Thirty-nine of 538 patients (7.2%, 95% confidence interval [CI] 5.2–9.2%) had positive blood cultures. In only one patient did the result of the blood culture definitively alter therapy. This patient had a fever of unknown origin, and the finding of a positive blood culture ultimately led to the diagnosis of chorioamnionitis. The mean duration of febrile morbidity was not significantly different in bacteremic vs. nonbacteremic patients (2.03 vs. 1.74 days). None of the repeat blood cultures was positive. The cost of blood cultures in the study population was $72,759.00.

*Conclusions:* The routine use of blood Cultures in the assessment of patients with chorioamnionitis rarely provides information that justifies a change in clinical management when patients are treated in accordance with the specific antibiotic protocol outlined in this investigation.


Infectious Diseases in Obstetrics and Gynecology 2:111–114 (1994)

© 1994 Wiley-Liss, Inc.

### Assessment of the Value of Routine Blood Cultures in the Evaluation and Treatment of Patients With Chorioamnionitis

**Gregory J. Locksmith and Patrick Duff**

*Division of Maternal-Fetal Medicine, Department of Obstetrics and Gynecology, University of Florida, Gainesville, FL*

### Abstract

*Objective:* The objective of this investigation was to determine the usefulness of blood cultures in evaluating patients with chorioamnionitis who were treated in accordance with a specific antibiotic protocol.

*Methods:* We reviewed the records of 539 patients with chorioamnionitis who delivered at our facility over a 3 year period (July 1, 1989–June 30, 1992). Patients had one set of aerobic and anaerobic blood cultures at the time of their initial assessment. They were treated initially with ampicillin or vancomycin plus gentamicin. Those who required cesarean delivery also received clindamycin postoperatively. Patients who had a poor initial response to therapy were treated empirically with selected antibiotics targeted against likely resistant organisms until the results of bacteriologic cultures were available. Bacteremic patients had repeat blood cultures while on therapy. We analyzed the medical records to determine the frequency with which blood culture results led to meaningful changes in patient management. We also compared the duration of febrile morbidity in bacteremic vs. nonbacteremic patients.

*Results:* Thirty-nine of 538 patients (7.2%, 95% confidence interval [CI] 5.2–9.2%) had positive blood cultures. In only one patient did the result of the blood culture definitively alter therapy. This patient had a fever of unknown origin, and the finding of a positive blood culture ultimately led to the diagnosis of chorioamnionitis. The mean duration of febrile morbidity was not significantly different in bacteremic vs. nonbacteremic patients (2.03 vs. 1.74 days). None of the repeat blood cultures was positive. The cost of blood cultures in the study population was $72,759.00.

*Conclusions:* The routine use of blood cultures in the assessment of patients with chorioamnionitis rarely provides information that justifies a change in clinical management when patients are treated in accordance with the specific antibiotic protocol outlined in this investigation.
© 1994 Wiley-Liss, Inc.

Key words

Bacteremia, intra-amniotic infection, cost effectiveness, outcome analysis, intrapartum infection

Chorioamnionitis is a common and serious complication of labor and delivery, occurring in 5–10% of patients.[1,2] Complications associated with chorioamnionitis include increased likelihood of cesarean delivery, neonatal sepsis and pneumonia, and persistent postpartum infection.[3–6]

Maternal bacteremia occurs in 5–10% of patients with chorioamnionitis. Blood cultures are frequently recommended to guide antibiotic therapy. On initial consideration, maternal bacteremia would seem to indicate a more severe infection, requiring longer administration of antibiotics and perhaps repeat blood cultures later in the course of the illness to ensure that serious sequelae of bacteremia do

Address correspondence/reprint requests to Dr. Patrick Duff, Department of Obstetrics and Gynecology, University of Florida College of Medicine, P.O. Box 100294, Gainesville, FL 32610-0294.

Clinical Study

Received May 10, 1994

Accepted August 1, 1994

not ensue.[7–11] However, the clinical utility of routine blood cultures in this clinical setting has never been assessed systematically.

Our objective in this study was to determine the usefulness of blood cultures in evaluating patients with chorioamnionitis who were treated in accordance with a well-defined antibiotic protocol. Specifically, we sought to determine how frequently blood culture results led to meaningful changes in patient management.

### MATERIALS AND METHODS

Using a computerized database, we identified the medical records of all patients with chorioamnionitis delivered at Shands Hospital-University of Florida from July 1, 1989, to June 30, 1992. During this period, 538 patients had a confirmed clinical diagnosis of chorioamnionitis and had at least one set of aerobic and anaerobic blood cultures at the time of their initial assessment. The clinical diagnosis of chorioamnionitis was based on a maternal fever of at least 37.8°C, maternal and fetal tachycardia, and uterine tenderness in the absence of any other localizing sign of infection.

Once the diagnosis was established, the patients were treated initially with IV ampicillin (2 g q 6h) and gentamicin (1.5 mg/kg q 8 h). Those with a history of penicillin allergy received gentamicin (1.5 mg/kg q 8 h) and clindamycin (900 mg q 8 h). In patients receiving ampicillin plus gentamicin who had a cesarean delivery, clindamycin (900 mg q 8 h) was added to the antibiotic regimen immediately postoperatively.[5] Patients undergoing cesarean delivery who became febrile just prior to surgery were treated with clindamycin plus gentamicin immediately after the infant's umbilical cord was clamped.

If a patient receiving ampicillin plus gentamicin failed to defervesce within 48 h, clindamycin was empirically added to the antibiotic regimen to enhance coverage of anaerobic organisms. If a patient receiving clindamycin plus gentamicin failed to defervesce within 48 h, ampicillin or vancomycin was administered to provide coverage of enterococci. Antibiotic therapy was continued until the patient had been afebrile for at least 24 h. Patients with bacteremia had repeat blood cultures at the time the initial cultures were reported as positive and were then arbitrarily continued on parenteral antibiotics until the second set of cultures was negative at 48 h of incubation. This practice was based upon our concern with the theoretical possibility of serious sequelae such as breakthrough bacteremia, septic shock, and abscess formation in patients who had positive blood cultures.[7,8,11]

We analyzed the medical records to determine the prevalence and microbiology of bacteremia and the clinical outcome of bacteremic patients. Specifically, we examined alterations in the selection of individual antibiotics, results of repeat blood cultures after apparent clinical cure, and causes for failure of antibiotic therapy. We also compared the duration of febrile morbidity in bacteremic vs. nonbacteremic patients. The nonbacteremic patients were selected from the study population using computer-generated random numbers.

We analyzed our data with the unpaired, two-tailed t-test. $P < 0.05$ was considered statistically significant.

### RESULTS

Of the 538 patients studied, 39 (7.2%, 95% confidence interval [CI] 5.2–9.2%) had positive blood cultures at the time of diagnosis. In 13 additional cases (2.4%, 95% CI 1.2–3.6%), coagulase-negative staphylococci or diphtheroid organisms were cultured from the blood and were considered to be skin contaminants. The most common pathogenic organisms were group B streptococci (15), *Escherichia coli* (9), anaerobic organisms (5), group A streptococci (4), and group D streptococci (3).

Of the 39 bacteremic patients, 7 had a change in their antibiotic coverage. In 4 cases, the change in therapy was based on a lack of clinical response to the initial therapy. Two of these patients had been diagnosed with chorioamnionitis just prior to undergoing cesarean delivery and were treated with clindamycin plus gentamicin after clamping of the infant's umbilical cord. Ampicillin was empirically added to the antibiotic regimen 24–36 h later because of persistent high fever. One patient subsequently had a blood culture positive for enterococci, but the other patient's blood culture grew group B streptococci which was sensitive to clindamycin. Another patient was placed on ampicillin plus gentamicin during labor and delivered vaginally. She continued to have a high fever 24 h postpartum so clindamycin was empirically added to the antibiotic regimen. Her blood culture was subsequently positive for *Peptostreptococcus* species. The final patient was discharged home afebrile after receiving ampicillin plus gentamicin for 24 h postpartum. She was readmitted 24 h after discharge with recurrent fever and empirically treated with ampicillin plus clindamycin plus gentamicin. Her aerobic blood culture was positive for gram-negative rods, but exact identification of the organism was not possible. She subsequently defervesced after 4 days of treatment.

In 3 cases, the change in therapy was based on blood culture results. In one of these patients, ampicillin was added to a clindamycin plus gentamicin regimen when the blood culture was found to have gram-positive cocci in pairs and chains. The organism was eventually identified as group B streptococci which was sensitive to the original antibiotic, clindamycin. Therefore, in retrospect, the addition of ampicillin was unnecessary. Another patient was initially placed on ampicillin and gentamicin, and *Klebsiella pneumoniae* was cultured from the blood. The organism was sensitive to both ampicillin and gentamicin, but the coverage was changed to ceftriaxone because the patient developed a coexistent pneumonia and an internal-medicine consultant recommended this change. Thus, in this instance, the blood culture result was not the decisive factor in altering antibiotic therapy. One patient presented to labor and delivery at 39 weeks with intact membranes, fever, and leukocytosis. The source of her infection was initially unknown, so cultures were taken from her blood, urine, and amniotic fluid. Although all cultures were subsequently positive, the blood culture was the first specimen to grow group B streptococci, and the diagnosis of chorioamnionitis was based on this result. The patient was then treated with antibiotics, and her delivery was expedited. Among the bacteremic patients, 28 had repeat blood cultures at the time the initial cultures were reported as positive. All repeat blood cultures were negative. In no instance, then, did the repeat cultures alter therapy.

The mean duration of fever in the bacteremic patients was 2.03 days, and the median was 1 day. For the nonbacteremic patients, the mean and median durations of febrile illness were 1.74 days and 1 day, respectively. These observed differences were not statistically significant. The inpatient cost for a set of aerobic and anaerobic blood cultures at our center is $118.50. Thus, the total cost of blood cultures in our study population was $72,759.00.

### DISCUSSION

The rationale for routinely obtaining blood cultures in patients with chorioamnionitis is to guide the selection of antibiotics and to identify more seriously ill patients who may require longer therapy.[7–11] Because of the polymicrobial nature of chorioamnionitis, many different pathogens may be isolated from bacteremic patients.[12] In our study, we recovered 12 different organisms in 39 positive blood cultures.

At our center, patients with chorioamnionitis are typically placed on 1 of 3 antibiotic regimens: ampicillin plus gentamicin, clindamycin plus gentamicin, or ampicillin plus clindamycin plus gentamicin. Any of these combinations should adequately treat infection caused by group A or B streptococci, anaerobic streptococci, *E. coli*, and most strains of staphylococci. A possible cause for concern is the patient with an enterococcal bacteremia being treated with gentamicin and clindamycin or the patient with an anaerobic gram-negative bacillus in the blood who is receiving ampicillin and gentamicin. Gram-negative aerobic bacilli that are resistant to aminoglycosides are unlikely to be encountered except in immunocompromised patients.

In only one case did the initial blood culture results definitively change therapy, and this instance was a patient with a fever of undetermined origin. The blood culture result was instrumental in establishing the diagnosis of chorioamnionitis in that case. There were 3 instances in which patients with enterococcal or anaerobic bacteremia were inadequately treated with the initial antibiotic therapy. In each of these cases, a lack of clinical response to therapy was noted before the blood culture results became available, and the antibiotic coverage was appropriately broadened. The 2 isolates of *Staphylococcus aureus* were sensitive to both gentamicin and clindamycin; in each case, the patients defervesced within 2 days of starting therapy.

None of the repeat blood cultures was positive.

Thus, in view of the comparable duration of febrile morbidity in bacteremic vs. nonbacteremic patients, the extended duration of antibiotic therapy in the former may not have been necessary. MacMillan and Grimes[13] have similarly demonstrated the limited usefulness of blood and urine cultures in treating pregnant patients with pyelonephritis.

Therefore, based on our findings, we conclude that the routine use of blood cultures does not improve diagnostic certainty in evaluating patients with chorioamnionitis who are treated in accordance with the antibiotic protocol used in this investigation. We acknowledge that none of the bacteremic patients in our study were immunosuppressed and none had underlying cardiac disease predisposing them to endocarditis. Blood cultures certainly are indicated in patients with heart disease who are at risk for endocarditis, in IV-drug abusers, in patients who have prosthetic heart valves or grafts, and in patients who are immunocompromised or who have an uncertain diagnosis. Additionally, blood cultures may be useful in evaluating patients who have a poor response to the initial antibiotic therapy or who may be at risk for having a highly resistant microorganism because of prolonged hospitalization or antibiotic therapy. However, one of these conditions is likely to be present in no more than 10% of patients. By using blood cultures more selectively in evaluating women with chorioamnionitis, obstetricians can simplify plans of management and lower treatment costs without compromising patient care.

### REFERENCES

1. Newton ER, Prihoda TJ, Gibbs RS: Logistic regression analysis of risk factors for intra-amniotic infection. Obstet Gynecol 73:571–575, 1989.
2. Soper DE, Mayhall CG, Dalton HP: Risk factors for intraamniotic infection: A prospective epidemiologic study. Am J Obstet Gynecol 161:562–568, 1989.
3. Sperling RS, Ramamurthy RS, Gibbs RS: A comparison of intrapartum versus immediate postpartum treatment of intra-amniotic infection. Obstet Gynecol 70:861–865, 1987.
4. Sperling RS, Newton E, Gibbs RS: Intra-amniotic infection in low-birthweight infants. J Infect Dis 157:113–117, 1988.
5. Gibbs RS, Duff P: Progress in pathogenesis and management of clinical intraamniotic infection. Am J Obstet Gynecol 164:1317–1326, 1991.
6. Duff P: Pathophysiology and management of postcesarean endomyometritis. Obstet Gynecol 67:269–276, 1986.
7. Duff P, Gibbs RS: Maternal sepsis. In Berkowitz RL (ed): Critical Care of the Obstetric Patient. New York: Churchill Livingstone, pp 189–217, 1983.
8. DiZerega GS, Yonekura ML, Keegan K, Roy S, Nakamura R, Ledger W: Bacteremia in post-cesarean section endomyometritis: Differential response to therapy. Obstet Gynecol 55:587–590, 1980.
9. Blanco JD, Gibbs RS, Castaneda YS: Bacteremia in obstetrics: Clinical course. Obstet Gynecol 58:621–625, 1981.
10. Ledger WJ, Norman M, Gee C, Lewis W: Bacteremia on an obstetric-gynecologic service. Am J Obstet Gynecol 121:205–212, 1975.
11. Kreger BE, Craven DE, Carling PC, McCabe WR: Gram-negative bacteremia. Am J Med 68:332–343, 1980.
12. Monif GRG, Baer H: Polymicrobial bacteremia in obstetric patients. Obstet Gynecol 48:167–169, 1976.
13. MacMillan MC, Grimes DA: The limited usefulness of urine and blood cultures in treating pyelonephritis in pregnancy. Obstet Gynecol 78:745–748, 1991.
